# Chronic Diarrhea in an Infant With Malrotation: A Diagnostic Dilemma

**DOI:** 10.1097/PG9.0000000000000177

**Published:** 2022-02-25

**Authors:** Rohit Josyabhatla, Nina Tatevian, Amanda S. Tchakarov, Charles S. Cox, Melissa R. Van Arsdall

**Affiliations:** From the * Department of Pediatrics, Division of Pediatric Gastroenterology, UTHealth McGovern Medical School, Houston, TX; † Department of Pathology and Laboratory Medicine, UTHealth McGovern Medical School, Houston, TX; and; ‡ Department of Pediatric Surgery, UTHealth McGovern Medical School, Houston, TX.

**Keywords:** chronic diarrhea, malrotation, very early onset inflammatory bowel disease, sucrose deficiency, *Clostridium difficile* infection

## Abstract

In children, diarrhea has a global incidence of 2.7 episodes per child-year and contributes to significant disease burden and mortality in children under 5 years of age. Chronic diarrhea, defined as diarrhea lasting for more than 2 weeks, may be particularly challenging to evaluate and manage in children under 2 years of age. While most have infectious enteritis or cow milk protein intolerance, others have conditions such as malnutrition, anatomic abnormalities, or congenital enteropathies that can be challenging to diagnose and treat. We present here a complex case of chronic diarrhea in an infant and highlight such diagnostic and therapeutic challenges.

## INTRODUCTION

Chronic diarrhea (CD) in an infant has a broad differential diagnosis. This case reviews one child’s challenging CD workup.

## CASE REPORT

A 4-month-old breastfed male presented, since birth, with poor weight gain, frequent spitting up, crying during defecation, and diarrhea (mucousy, nonbloody, voluminous, about 5 episodes daily). His weight was at the third percentile and weight-for-length at the first percentile. His symptoms persisted despite mother’s strict dietary elimination and his subsequent placement on amino acid-based formula. His diarrhea later worsened upon solid food introduction.

**FIGURE 1.**
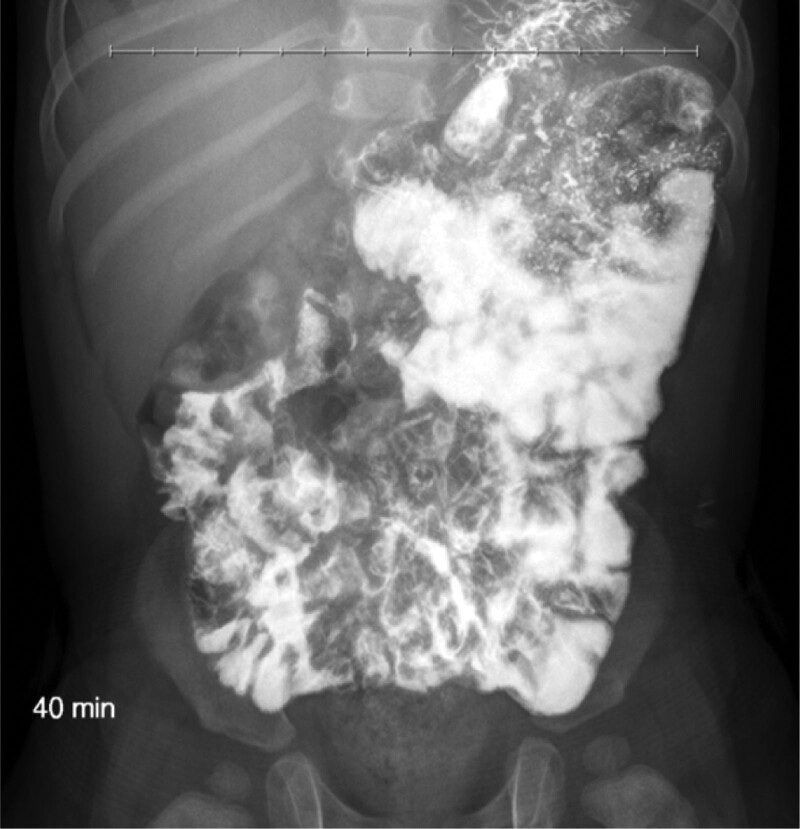
UGI image 40 minutes after oral contrast intake, with poor delineation of duodenojejunal junction. UGI = upper gastrointestinal.

Multiple, serial hemoccult stool tests were positive. Complete blood count, complete metabolic profile, lipase, sedimentation rate, immunoglobulin levels, and stool for reducing substances, pancreatic elastase, and calprotectin were normal. C-reactive protein was mildly elevated (10.3 mg/L). Barium enema showed short segmental narrowing at the rectosigmoid junction, but a normal rectosigmoid ratio. Rectal suction biopsy was normal. Multiple abdominal radiograph showed no obstruction. An upper gastrointestinal (UGI) series with small bowel follow-through was inconclusive due to poor contrast intake (Fig. 1).

**FIGURE 2.**
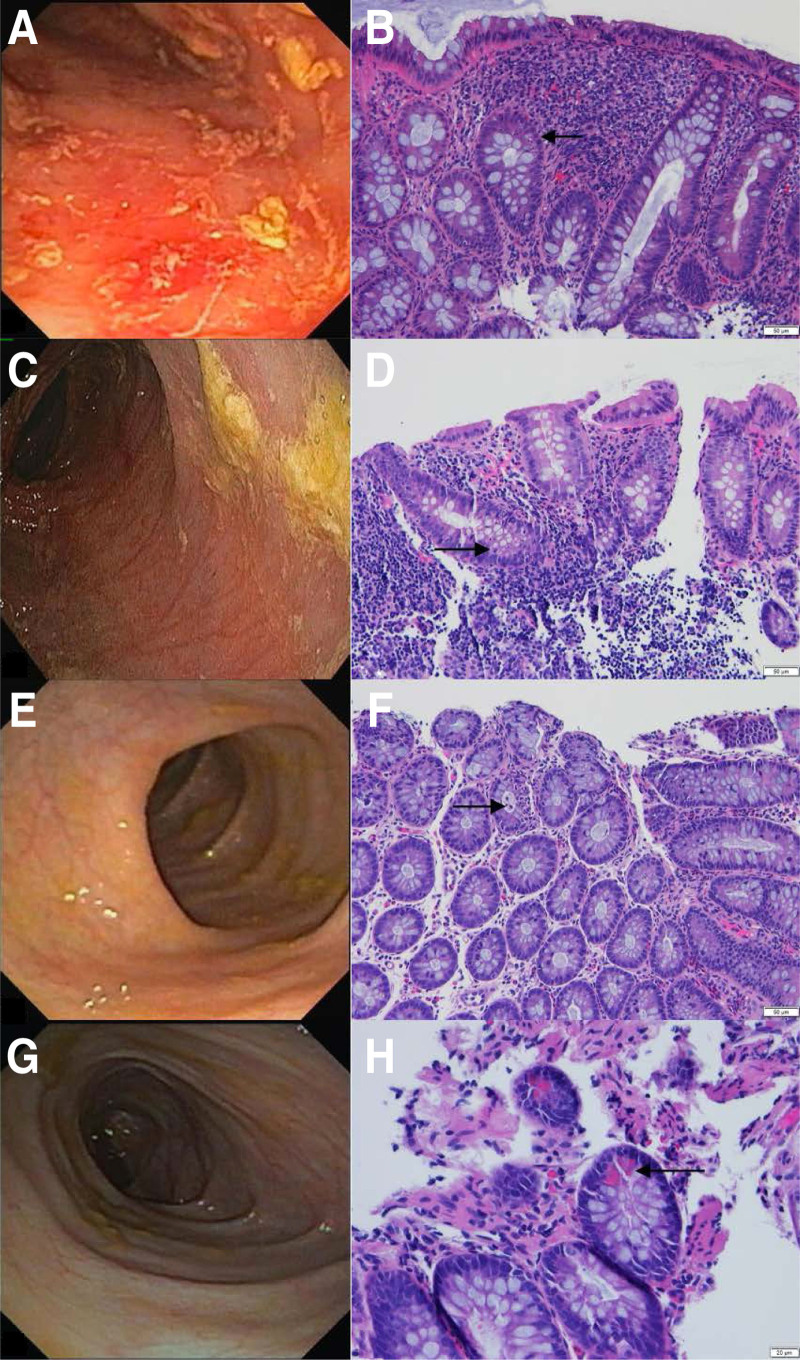
Colonoscopy and Histopathology images. (A, B) at age 11 months. (A) Edema, erythema, with superficial bleeding at 30 cm from anal verge (descending colon); (B) Mild crypt distortion and focal mild active cryptitis (arrow), 15 cm from anal verge. (C and D) at age 12 months. (C) Endoscopically normal descending colon; (D) Mild crypt distortion and focal mild active cryptitis (arrow) in the descending colon; (E and F) at age 16 months. (E) Endoscopically normal splenic flexure; (F) Mild crypt distortion and mild active cryptitis with focal small crypt abscess (arrow) in the descending colon; (G and H) at age 19 months. (G) Endoscopically normal descending colon; (H) Paneth cell metaplasia (arrow) in the descending colon.

Endoscopy at 11 months revealed marked inflammation at the splenic flexure; histopathology showed focal acute inflammation (Fig. 2A, B). Duodenal biopsies revealed deficiencies of sucrase (16.2, reference 25–69.9 µM/min/g protein) and isomaltase (undetectable). Other disaccharidase levels were normal—lactase (31.88, reference 15–45 µM/min/g protein) and maltase (123.46, reference 100–224 µM/min/g protein). Sacrosidase was started for sucrase deficiency. Prednisolone and sulfasalazine were started for the colonic inflammation, although subsequent very early onset inflammatory bowel disease (VEO-IBD) gene panel was unremarkable.

After 1 month of prednisolone, his colon was endoscopically normal, but biopsies showed active colitis (Fig. 2C, D). He then had 2 bouts of *C. difficile* infection (CDI) that complicated matters. Both episodes, 2 months apart, were characterized by acute worsening of his baseline diarrhea and abdominal pain and by positive PCR tests and toxin assays. He was first treated with metronidazole and then vancomycin. On both occasions, treatment resulted in partial improvement of his diarrhea with negative follow-up toxin assays, suggesting treatment response.

His sulfasalazine was then switched to mesalamine, which did not help. Colonoscopy at 16 months showed chronic active colitis (Fig. 2E, F). A trial of budesonide and later escalation of therapy to infliximab (IFX) seemed to partially improve his diarrhea but not his weight gain. Following IFX induction, his colonoscopy showed persistent chronic inflammation but no active inflammation (Fig. 2G, H). He was then tapered off budesonide and maintained on IFX. His diarrhea thereafter worsened, despite adequate infliximab levels and no anti-infliximab antibodies.

**FIGURE 3.**
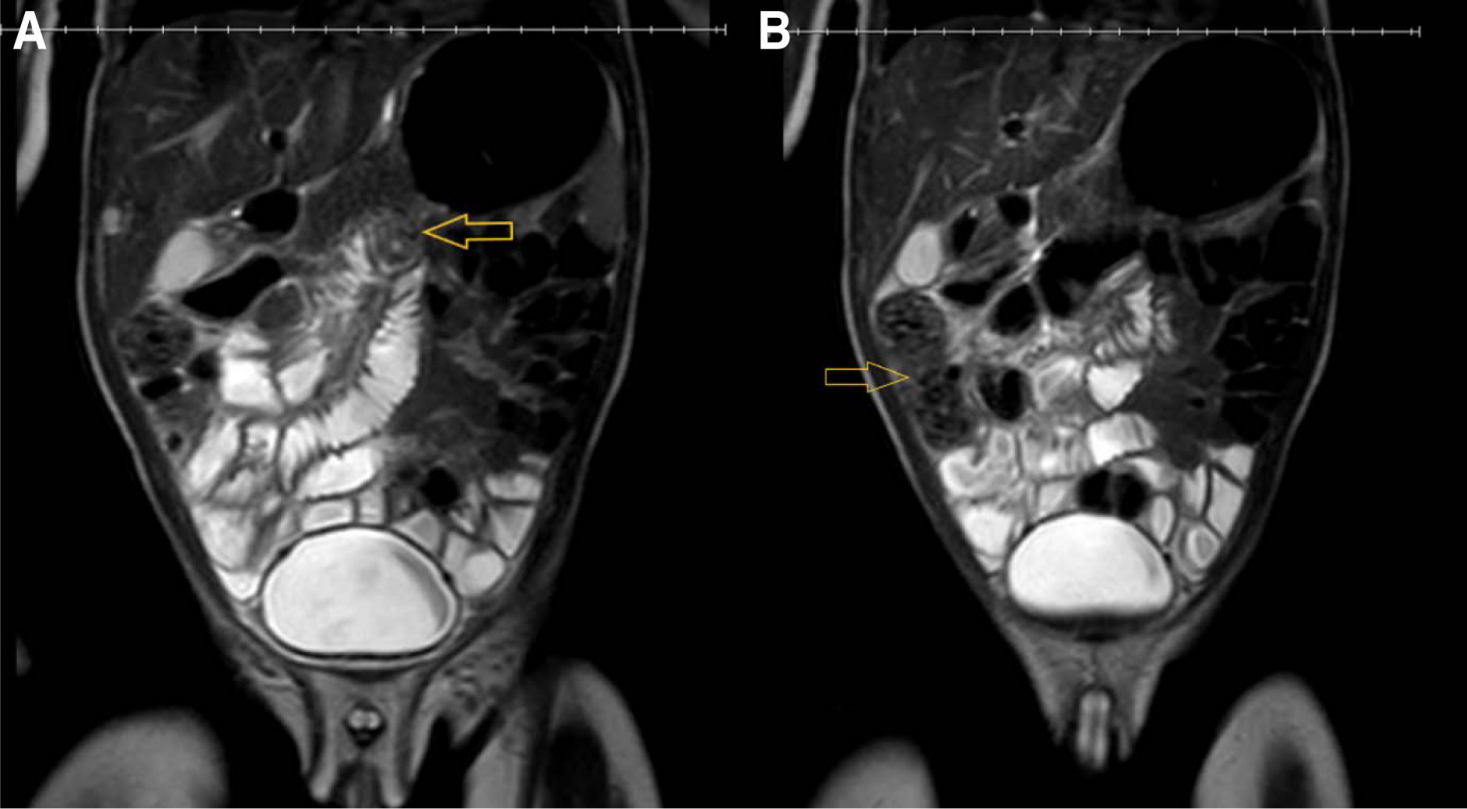
MRE images. (A) Yellow arrow indicating the displaced duodenojejunal junction; (B) Yellow arrow indicating the high riding cecum. MRE = magnetic resonance enterography.

Magnetic resonance enterography (MRE), previously delayed by coordination issues, showed intestinal nonrotation without evidence of inflammation (Fig. 3). Infliximab was then stopped, and he underwent a Ladd procedure. He has since remained symptom-free while only supplemented with sacrosidase for sucrase-isomaltase deficiency. One year after his surgery, all biopsies from follow-up EGD and colonoscopy were histologically normal.

## DISCUSSION

Malrotation, defined as failure of the normal rotation or fixation of any portion of the gastrointestinal tract, typically presents in the first month of life with abdominal distension and bilious vomiting, secondary to intestinal obstruction by peritoneal bands (Ladd’s bands) coursing from the cecum across the duodenum to the right abdominal wall. The most feared complication, midgut volvulus, results from twisting of the small intestine around a narrow mesenteric root.^1^ Atypical symptoms, usually occurring beyond infancy, include recurrent abdominal pain, malabsorption, CD, solid food intolerance, and poor weight gain, hypothesized to be secondary to chronic intermittent ischemia or lymphatic obstruction.^2^ Imamoglu et al described 4 patients (8 months to 10 years) presenting with malabsorption-like symptoms and chronic midgut volvulus secondary to intestinal malrotation; the median interval between symptom onset and diagnosis was 35 months.^3^ Our patient presented with atypical symptoms during infancy, highlighting the importance of considering malrotation in the differential diagnosis of failure to thrive and CD in an infant.

In malrotation, UGI series shows abnormal position of the duodenojejunal junction to the right of the vertebral column with 96% sensitivity. Barium enema may reveal an abnormal cecal position. While ultrasound (US) and computed (CT) scan may demonstrate a mesenteric whirlpool sign during volvulus, their utility in an asymptomatic patient is debated.^4,5^ Multiple abdominal radiographs showed no evidence of intestinal obstruction in our patient, and his UGI series was a limited study. MRE ultimately led to his diagnosis. Therefore, in a patient unable to ingest contrast adequately, use of a nasogastric tube to administer contrast or a more detailed study like MRE or CT should be promptly pursued.

There are limited reports of patients with both malrotation and IBD.^6–8^ Our patient’s constellation of clinical findings, histology consistent with chronic active inflammation, and partial improvement with steroids prompted concern for VEO-IBD. In retrospect, this was likely nonspecific inflammation secondary to chronic midgut volvulus that mimicked findings that are common to VEO-IBD, autoimmune enteropathy, and immune deficiency states.

Recent studies have reported increasing prevalence of congenital sucrase-isomaltase deficiency (CSID), with presence of symptoms even in heterozygous carriers.^9^ Stool reducing substances are negative in CSID. The duodenal/jejunal biopsy is not entirely specific. In a patient like ours, diarrhea after weaning from a milk-based diet should prompt CSID evaluation. Treatment includes sacrosidase enzyme supplementation.

*C. difficile* infection further confounded this patient’s later diagnosis of malrotation. Carriage rates for Clostridium difficile are high in infancy (30–37%) and decrease by 3 years (0–3%).^10^ On both occasions with CDI our patient had positive *C. difficile* toxin B cytotoxin assay results, clinical improvement with antibiotics, and negative cytotoxin assay results after treatment. Thus, we believed that his acutely worsened and then otherwise unexplained diarrhea with a positive toxin assay warranted treatment.

In conclusion, malrotation should be considered in the differential diagnosis of infants and children with CD and poor growth. UGI series is the gold standard but is sometimes difficult to perform and interpret in an uncooperative child. If inconclusive, ultrasound, MRE, or CTE may aid diagnosis. Most importantly, early diagnosis and surgery could help reduce potential adverse outcomes like short bowel syndrome.

## ACKNOWLEDGMENTS

An informed consent was obtained from the parent to publish this case report.
